# Dose-response of fibrinogen and factor XIII concentrate for correcting albumin-induced coagulopathy

**DOI:** 10.1186/cc12299

**Published:** 2013-03-19

**Authors:** US Schött, DW Winstedt, JH Hanna

**Affiliations:** 1 Lund University, Lund, Sweden

## Introduction

Natural colloid albumin induces a lesser degree of dilutional coagulopathy than synthetic colloids. Fibrinogen concentrate has emerged as a promising strategy to treat coagulopathy, and factor XIII (FXIII) works synergistically with fibrinogen to correct coagulopathy following haemodilution with crystalloids. Objectives were to examine the ability of fibrinogen and FXIII concentrates to reverse albumin-induced dilutional coagulopathy.

## Methods

High and low concentrations of both fibrinogen and FXIII were used to reverse coagulopathy induced by 1:1 dilution *in vitro *with 5% albumin of blood samples from healthy volunteers, monitored by rotational thromboelastometry (ROTEM).

## Results

Haemodilution with albumin significantly attenuated EXTEM maximum clot firmness (MCF), α angle (AA), clotting time (CT) and clot formation time (CFT), and FIBTEM MCF (*P <*0.001). Following haemodilution, both doses of fibrinogen significantly corrected all ROTEM parameters (*P *≤0.02), except the lower dose did not correct AA. Compared with the lower dose, the higher dose of fibrinogen significantly improved FIBTEM MCF and EXTEM MCF, AA and CFT (*P <*0.001). The lower dose of FXIII did not significantly correct any of the ROTEM parameters, and the high dose only improved EXTEM CT (*P *= 0.004). All combinations of high/low concentrations of fibrinogen/ FXIII significantly improved all ROTEM parameters examined (*P *≤0.001). Fibrinogen concentration generally had a greater effect on each parameter than did FXIII concentration; the best correction of ROTEM parameters was achieved with high-dose fibrinogen concentrate and either low-dose or high-dose FXIII.

## Conclusion

Fibrinogen concentrate successfully corrected initiation, propagation and clot firmness deficits induced by haemodilution with albumin, and FXIII synergistically improved fibrin-based clot strength.
